# Editorial: Mechanisms regulating immunity in plants

**DOI:** 10.3389/fpls.2013.00064

**Published:** 2013-03-27

**Authors:** Alexandra M. E. Jones, Jacqueline Monaghan, Vardis Ntoukakis

**Affiliations:** ^1^The Sainsbury Laboratory, Norwich Research ParkNorwich, UK; ^2^School of Life Sciences, University of WarwickCoventry, UK

**Figure 1:**
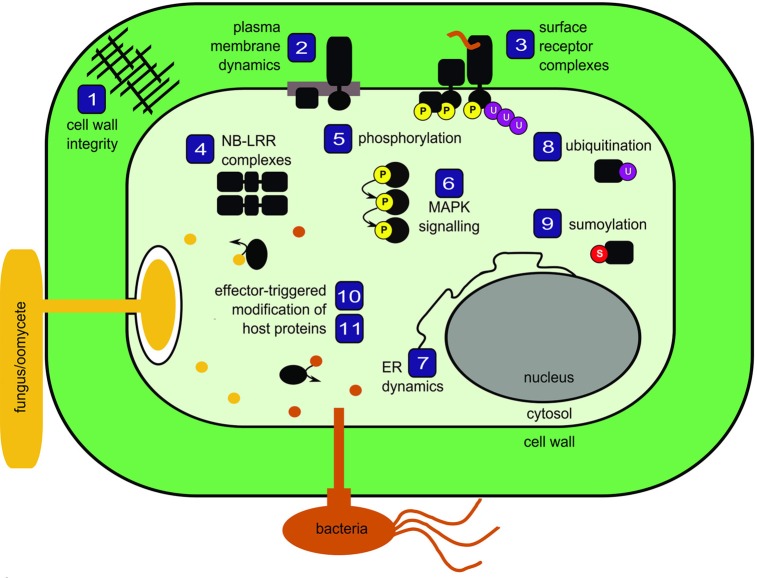
**Schematic representation of topics covered in the special issue *Mechanisms regulating immunity in plants*.** Numbers correspond to review articles as follows: (1) Nühse (2012), Cell wall integrity signaling and innate immunity in plants; (2) Urbanus and Ott (2012), Plasticity of plasma membrane compartmentalization during plant immune responses; (3) Greeff et al. (2012), Receptor-like kinase complexes in plant innate immunity; (4) Bonardi and Dangl (2012), How complex are intracellular immune receptor signaling complexes? (5) Park et al. (2012), Protein phosphorylation in plant immunity: insights into the regulation of pattern recognition receptor-mediated signaling; (6) Rasmussen et al. (2012), MAP kinase cascades in *Arabidopsis* innate immunity; (7) Eichmann and Schäfer (2012), The endoplasmic reticulum in plant immunity and cell death; (8) Furlan et al. (2012), Regulation of plant immune receptors by ubiquitination; (9) Mazur and van den Burg (2012), Global SUMO proteome responses guide gene regulation, mRNA biogenesis, and plant stress responses; (10) Wirthmueller and Banfield (2012), mADP-RTs: versatile virulence factors from bacterial pathogens of plants and mammals; (11) Howden and Huitema (2012), Effector-triggered post-translational modifications and their role in suppression of plant immunity.

Plants are constantly exposed to potential pathogens in their environment. The intimate associations involved in plant-microbe interactions have influenced the evolution of a multi-faceted surveillance system to detect and respond to both the presence of microbes at the cell surface as well as the presence of pathogenic effectors inside the cell. Here, we bring together 11 reviews that discuss current concepts in plant innate immunity with a focus on protein biology and proteomics (Figure 1).

To interact with the plant plasma membrane, microbes must first breach the formidable barrier presented by the cell wall. Nühse (2012) introduces the emerging concept of cell wall integrity signaling, noting that both mechanical properties and receptors capable of sensing cellular damage are likely to be involved. In both pathogenic and symbiotic interactions with microbes, the host plasma membrane is substantially modified. Urbanus and Ott (2012) review the dynamic compartmentalization of the plasma membrane and discuss factors, such as alterations to lipid composition and/or anchoring of proteins to the cell wall or cytoskeleton, that contribute to the formation of membrane micro-domains. Embedded within the plasma membrane, pattern recognition receptors (PRRs) can be affected by the formation of micro-domains. Many PRRs are receptor-like kinases that bind ligands derived from microbes and, as discussed by Greeff et al. (2012), rapidly form complexes with other proteins to initiate signaling cascades. Within the plant cell, additional immune receptor complexes detect the presence of pathogenic effector proteins. Bonardi and Dangl (2012) describe pre- and post-activation mechanisms regulating intracellular receptor complexes and recognize the need to use emerging fluorescent protein technologies in parallel to proteomics in order to study spatio-temporal dynamics of immune receptors in living cells.

Of all the molecular events that occur within activated receptor complexes, the most intensely studied using proteomic methods is phosphorylation, both for the amenability of this modification to analysis and for the central role it plays in signal transduction in all organisms. Park et al. (2012) nicely review the role of phosphorylation in all stages of immune signal transduction downstream of PRRs. The authors identify the need to clarify *in vivo* phosphorylation events and they highlight the continued gap in our knowledge between activated receptor complexes and downstream signaling cascades, such as those mediated by mitogen-activated protein kinases, which are discussed in detail by Rasmussen et al. (2012).

The endoplasmic reticulum (ER) is closely connected to defense responses, both as a large intracellular store of calcium and as the site of immune receptor biogenesis. Eichmann and Schäfer (2012) review the integration of stress responses by the ER and its role in initiating programmed cell death through the activation of ER-resident regulatory proteins, drawing comparisons with better characterized animal systems. In addition to ER folding machinery, the turn-over of both plasma membrane-localized and intracellular receptors relies upon ubiquitination. Furlan et al. (2012) review these aspects and explore proteomic methods to identify novel ubiquitination sites. Recent progress has also been made in proteomics to identify modifications by the small ubiquitin-like protein SUMO. Encouragingly, Mazur and van den Burg (2012) describe the use of histidine-tagged SUMO as “routine” and compare proteins identified by these and more advanced methods in plants and animals in the context of SUMO dynamics in abiotic and biotic stress responses.

Adapted pathogens must evade or suppress host immune responses in order to colonize tissues and cause disease, and they deploy numerous effector proteins to secure this objective. Wirthmueller and Banfield (2012) focus on pathogenic mono ADP-ribosyltransferases as important virulence factors acting on host targets in both plant and animal systems. Given the importance of post-translational modifications of proteins in the plant immune system, Howden and Huitema (2012) explore how pathogen effectors modify the post-translational status of host proteins to interfere with defense signaling. The authors also offer insight into experimental approaches for effector/target mining.

Proteomic methods have facilitated the identification of key players involved in plant immunity and have shed light on the significance of post-translational modifications and protein interactions in the regulation and transduction of immune signaling. In future, the use of large-scale and highly sensitive quantitative proteomics in combination with emerging transcriptomic and imaging technologies will play a central role in uncovering the kinetics of immune signaling pathways, which currently remains a challenge. This is an exciting time to be involved in plant immunity research and we hope that this collection of reviews will inform and inspire our readers.
